# Gemcitabine Plus Carboplatin in Patients with Advanced Hepatocellular Carcinoma: Results of a Phase II Study

**DOI:** 10.5402/2012/420931

**Published:** 2012-07-16

**Authors:** Aly M. Azmy, Khalid E. Nasr, Nagy S. Gobran, M. Yassin

**Affiliations:** Clinical Oncology Departement, Faculty of Medicine, Ain Shams University, Cairo, Egypt

## Abstract

*Objectives*. Assessment of gemcitabine/carboplatin combination in patients with advanced-stage hepatocellular carcinoma (HCC) in a phase II trial for safety and efficacy. *Methods*. Forty patients with previously untreated advanced-stage HCC were prospectively enrolled and subjected to gemcitabine/carboplatin regimen which consisted of gemcitabine 1000 mg/m^2^ on days 1 and 8, and carboplatin AUC 6 on day 1. The treatment was repeated every 3 weeks until disease progression or limiting toxicity. *Results*. Forty patients were assessable for efficacy and toxicity. In all, 276 treatment cycles were administered. No toxic deaths occurred. Hematological grade 3-4 toxicity consisted of thrombocytopenia (27% of patients) and neutropenia (24%), including 2 febrile neutropenia and anemia (9%). Grade 3 carboplatin-induced neurotoxicity was observed in 3 (9%) patients. ORR was 23% (95% CI, 0.10–0.29) with 9 partial responses and disease stabilization was observed in 46% (95% CI, 0.22–0.42) of patients, giving a disease control rate of 69%. Median progression-free and overall survival times were, respectively, 5 months (95% CI: 3–8 months) and 8 months (95% CI: 6–18 months). *Conclusion*. The gemcitabine/carboplatin regimen seems to be effective, well tolerated, and active in advanced HCC.

## 1. Introduction

Hepatocellular carcinoma is the fifth most common cancer in men and eighth in women worldwide, resulting in at least 500,000 deaths per year [1]. Over a decade, there was nearly a twofold increase of the proportion of HCC among CLD patients in Egypt with a significant decline of HBV and slight increase of HCV as risk factors. Alpha-fetoprotein played a limited role in diagnosis of HCC, compared to imaging techniques [2].

Diagnosis is usually made by history, physical examination, imaging (US, CT, MRI), and elevated serum AFP > 400 ng/mL with 75% of hepatocellular carcinoma is multifocal at time of diagnosis. In most patients with HCC, we are dealing with two independent diseases, each determines the patient outcome. Treatment plan should consider the disease extent, hepatic functional reserve, and patient's performance status [3].

Liver resection is the first curative option with 3 yr survival of 54% in the noncirrhotic liver after R0 resection. Transplantation comes next in patients fulfilling Milan criteria, or the expanded UCSF criteria, with 3 yr survival of up to 88%. Ablative modalities such as TACE, RFA, and others are accepted alternatives either as palliation, or bridging before liver transplant. For HCC patients with extrahepatic extent or extensive disease not fit for surgery, systemic therapy is the only choice. Until recently there has been no standard medical therapy for advanced HCC. Sorafenib was the first to show significant impact on survival and disease progression, and is widely accepted as a standard first-line systemic therapy [4].

Due to financial cost of sorafenib, and need to improve response and survival, the need to search for other nonhepatotoxic regimens of systemic therapy for HCC is investigated. Results obtained in phase II studies with different regimens using new cytotoxic drugs have not been very impressive. Thus, systemic chemotherapy cannot be considered as the standard of care for HCC patients. This situation could be related to a combination of poor efficacy and increased toxicity with underlying liver cirrhosis. Also hepatitis B virus reactivation after chemotherapy-induced immunodepression, producing an additive toxic effect [5]. Systemic chemotherapy likely lacks efficacy because of the frequently observed multidrug tumor resistance (P-glycoprotein overexpression, p53 gene mutations) [6, 7].

## 2. Patients and Methods

Patients were eligible if they had advanced-stage HCC not amenable to curative treatment. HCC had to be pathologically documented, or to meet the following criteria: *α*-fetoprotein (AFP) level over 400 ng/mL, together with a hypervascular liver tumor and cirrhosis; measurable disease according to the RECIST system, with at least one lesion measuring at least 2 cm on computed tomography (CT) or magnetic resonance imaging (MRI) performed <20 days before accrual; documented progressive disease on 2 consecutive CT scans and/or MRI performed at a 2-month interval, or clinical progression according to RECIST; compensated Child-Pugh stage A or B cirrhosis, score < 9; no previous systemic chemotherapy or radiotherapy for HCC; age at least 18 years; World Health Organization (WHO) performance status (PS) of 0 to 2, adequate blood cell counts (neutrophils > 1.5 × 109/L and platelets > 100 × 109/L), and renal function (s.creatinine < 1.4 mg/dL) within the 2 weeks before study entry.

 Exclusion criteria included known central nervous system metastases; human immunodeficiency virus (HIV) infection; an interval shorter than 8 weeks since transarterial chemoembolization (if performed); history of sensory peripheral neuropathy; alkaline phosphatase > 5 times the upper normal limit (UNL), INR < 60%, serum albumin < 3.0 g/L, and bilirubin ≥ 1.5 UNL.

*Pretreatment investigations* included a complete medical history and physical examination, AFP assay, electrocardiogram, hematologic and biochemical profiles, abdominal CT or MRI, and thoracic CT in case of suspected lung metastases. Body weight, PS, and clinical manifestations were recorded before the outset of therapy. The study was conducted in Ain Shams university hospitals through the period from June 2009 to March 2011. Written consents were obtained from the patients before enrollment in the study.

### 2.1. Treatment Protocol and Dose Modification

Chemotherapy consisted of gemcitabine at a dose of 1000 mg/m^2^ as a fixed dose rate intravenous infusion of 10 mg/m^2^/minute on days 1 and 8, followed by carboplatin AUC 6 as a 2-hour infusion on day 1. Treatment was repeated every 3 weeks. If grade 3/4 (nonneurosensory) toxicity occurred the subsequent cycle was postponed until recovery to grade <2; the gemcitabine dose was then reduced to 800 mg/m^2^ and the carboplatin dose to AUC 5. If grade 3 cumulative sensory peripheral neuropathy occurred, carboplatin was discontinued and gemcitabine was administered alone as initially scheduled. Preventive calcium and magnesium infusions were used to reduce the risk of neurotoxicity. Antiemetic prophylaxis was done routinely before infusion. Treatment was continued until disease progression, unacceptable toxicity, patient refusal, or until chemotherapy had to be delayed for more than 3 weeks because of toxicity.

### 2.2. Response Assessment

The primary endpoint for efficacy was the objective response rate (ORR), defined as the sum of complete and partial responses based on the RECIST criteria version1.1. Tumor responses were assessed by means of MRI or helical CT according to the initial diagnosis method, every 2 months (after 4 cycles), or earlier in patients with suspected disease progression. A second MRI or CT scan was performed 4 and 8 weeks after the first to confirm response. Secondary endpoints for efficacy included progression-free (PFS) and overall survival (OS) times. AFP levels were measured every 2 months. Body weight, PS, and symptoms were recorded before each cycle.

### 2.3. Toxicity Assessment

Toxicity was graded according to the National Cancer Institute's Common Toxicity Criteria (NCI-CTC; version 4.0, December 2010), based on clinical and biologic findings before each treatment cycle, then at the end of treatment and one month later. The patients were interviewed before each session, focusing on pain, nausea, vomiting, mucositis, diarrhea, asthenia, weight loss, and dermatologic and neurologic disorders. All patients who received at least one dose of study treatment were evaluated for toxicity.

### 2.4. Statistical Analysis

The primary endpoint was the ORR, and its exact 95% confidence interval (95% CI). The 1-sample multiple testing procedure for phase 2 clinical trials was used to calculate the sample size [8]. On the basis of an anticipated ORR of 25% for gemcitabine and carboplatin, and the best rates obtained in recent trials (approximately 10%), a total of 39 patients were required with *α* = 5% and *β* = 20%. The secondary efficacy endpoints were the disease control rate (DCR: CR + PR + SD), changes in the AFP plasma level, and PFS and OS. The toxicity analysis was based on the worst grade in each patient during any chemotherapy cycle. All analyses were performed on an intention-to-treat (ITT) basis. The results were expressed as the mean ± standard deviation, or range, as appropriate. Followup started at the first dose of study treatment. The censoring event for responses was the onset of disease progression. The censoring event for survival was the date of death or lost follow up. Survival curves were plotted with GraphPad prism version 4.03 using the Kaplan-Meier method.

## 3. Results

### 3.1. Patient Characteristics

Between June 2009 and March 2011, 40 eligible patients with unresectable or metastatic HCC were enrolled and treated at Ain Shams university hospitals, Clinical Oncology Department. Patient characteristics are shown in Table 1.

### 3.2. Efficacy

**Figure 1:**
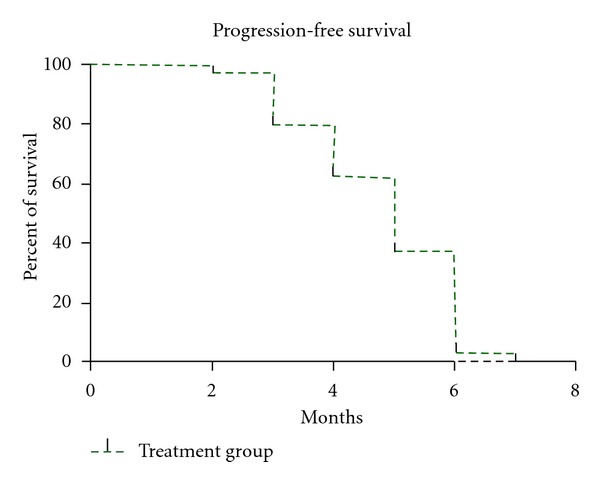
Progression-free survival (PFS) (dashed line) in the intent-to-treat population (*n* = 40).

**Figure 2:**
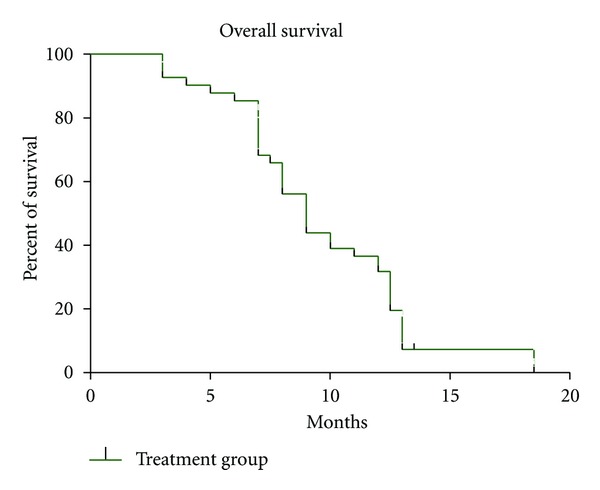
Overall survival (OS) (solid line) in the intent-to-treat population (*n* = 40).

ORR was 23% (95% CI, 0.10–0.29); pattern of response is shown in Table 2. The responses lasted from 3 to 12 months. Stable disease was observed in 46% (95% CI, 0.22–0.42) of patients, in assessable patients who had at least 1 postbaseline tumor assessment (*n* = 40) the disease control rate was 69% (PR, 23% and SD, 46%). In the ITT population, the median PFS was 5 months (95% CI, 3–8 months) (Figure 1), and the median OS was 8 months (95% CI, 6–18 months) (Figure 2). The 1-year survival rate was 35.4%. The AFP level fell by >50% during therapy in 12 (38%) of the 31 patients with elevated AFP levels at baseline (>400 ng/mL), of which 5 patients had partial response and 7 patients had SD.

### 3.3. Treatment Exposure

Overall, 276 cycles of treatment were administered to the 40 patients, with a mean dose delivered intensity 80% for gemcitabine, and 85% for carboplatin, with a mean of 5 cycles per patient (range, 1–8 cycles). The chemotherapy dosage was reduced in 10 patients (25%), because of hematologic toxicity (*n* = 10) or carboplatin-induced decreased GFR (*n* = 5), after a mean of 12 weeks on full treatment. Treatment had been discontinued in 39 patients; because of surgery in one patient (*n* = 1), disease progression (*n* = 30), adverse events (*n* = 4), and patient refusal to continue (*n* = 4).

### 3.4. Subsequent Treatments

Thirteen patients received subsequent treatments, ten patients received UFT, two patients had chemoembolization, and one patient received sorafenib.

### 3.5. Toxicity

Toxicity pattern is shown in Table 3. Myelosuppression, nephrotoxicity, and neurotoxicity were the most frequent adverse effects. No treatment-related deaths occurred.

## 4. Discussion

Gemcitabine and carboplatin are active agents against HCC. A combination of both is tested in this phase II study for response, and toxicity as primary outcome, and for impact on survival and disease progression as secondary outcome. Overall response rate was 23% (95% CI: 8–34). Stable disease was obtained in 46%, with disease control rate of 69%. Median progression-free and overall survival were, respectively, 5 months (95% CI: 3–8 months), and 8 months, (95% CI: 6–18).

Regarding toxicity assessment, no toxicity-related death occurred; however grade 3 and 4 were encountered as follows: neutropenia (40%), thrombocytopenia (17.5%), anemia (25%), alopecia (0%), diarrhea (0%), vomiting (2.5%), neurotoxicity (20%), nephrotoxicity (15%), and hepatotoxicity (15%).

Tumour response rates of 15–25% were previously obtained with doxorubicin and cisplatin combinations with either capecitabine or UFT. However, this did not seem to affect significantly PFS and OS found to be less than 4 months and 8 months, respectively [9, 10]. Response rate was similar to that in this study. Randomized phase III study comparing single-agent doxorubicin versus PIAF regimen (cisplatin/interferon a-2b/doxorubicin/fluorouracil) did not show any significant difference in OS between the two arms despite borderline statistical significance in favour of PIAF (6.8 and 8.7 months for doxorubicin and PIAF arms, resp.) [11]. Xelox regimen was assessed in phase II trial in 50 patients with HCC, best tumour response was partial response (PR) in 3 patients (7%), stable disease (SD) in 33 patients (81%), and disease progression in 5 patients (12%). Partial response duration in the three patients was 1.1, 5.0, and 7.3 months, respectively, whereas duration of SD ranged from 2.2 to 20.5 months (median: 5.4 months). In the intention-to-treat group (*N* = 50), the tumour control rate (PR and SD) was 72% (95% confidence interval (CI) 57–83%). The tumour control rate was 77% (95% CI 61–88%) in the 43 patients with Child-Pugh Scores for cirrhosis, including the three patients with PR Progression-free survival rates at 6 and 12 months were 38% (95% CI 26–52%) and 14% (95% CI 7–26%), respectively. Main grade 3-4 drug-related toxicities included diarrhea (16%), elevation of aminotransferases and/or bilirubin (16%), thrombocytopenia (12%), and neurotoxicity (6%) [12]. Tumour control rate and toxicity were comparable to that in our study. In another phase II trial assessing 41 patients with HCC subjected to gemcitabine and pegylated liposomal doxorubicin, the median TTP and OS were 5.8 and 22.5 months, respectively. Hematologic toxicity was the most common side effect, including neutropenia (17%) and anemia (7%) [13].

HCC patients given the ECF/ECC regimen obtained objective response rate 22%, with a disease control rate (objective response plus stable disease) of 52%. The median time to progression was 6 months. In addition, despite the fact that most tumors were huge, the reduction in tumor size was sufficient to allow surgical resection in 2 patients having only one huge tumor. Toxicity was mild and most side effects were manageable; one patient died suddenly between two courses. These two regimens (ECF and ECC) are very similar in terms of response and toxicity since capecitabine is the oral form of 5FU [5]. Response rate is close to that obtained from gemcitabine/carboplatin in this study.

In another phase II study assessing 45 patients with advanced-stage HCC treated with gemcitabine, oxaliplatin combined with cetuximab, the median progression-free and overall survival times were 4.7 months and 9.5 months, respectively. The 1-year survival rate was 40%. Grade 3 to 4 hematologic toxicity consisted of thrombocytopenia (24%), neutropenia (20%), and anemia (4%). Grade 3 oxaliplatin-induced neurotoxicity occurred in 5 patients (11%) and grade 3 cutaneous toxicity in 7 patients (16%). Results and toxicity profile were close to this study apart from cutaneous toxicity probably added by cetuximab [14].

In conclusion, gemcitabine and carboplatin is a safe and effective combination in management of advanced hepatocellular carcinoma not candidate for surgical resection or other interventional measures with fair control rate and accepted toxicity profile. Despite results are still inferior to sorafenib, the current standard of care, this regimen is an acceptable alternative if sorafenib is not available, or patient experienced failure or unaccepted toxicity from sorafenib.

## Figures and Tables

**Table 1:** Patient characteristics.

Characteristic	No. of patients (%)
No. of patients	40
Median age (range), y	55 (44–69)
Gender	
Male	37 (92.5%)
Female	3 (7.5%)
WHO performance status	
0	1 (2.5%)
1	30 (75%)
2	9 (22.5%)
Child-Pugh stage	
A	28 (70%)
B	12 (30%)
Median AFP (range), ng/mL	450 (3–99500)
AFP >400 ng/mL	31 (77.5%)
Histologic diagnosis of HCC	20 (50%)
Diagnosis of HCC based on Barcelona criteria	20 (50%)
Patients with extrahepatic disease	12 (30%)
Previous treatment	
None	15 (37.5%)
Radiofrequency ablation	10 (25%)
Curative surgery	3 (7.5%)
TACE	12 (30%)
Systemic therapy	0

**Table 2:** Response rate.

Response rate	No. (%)
Partial response	9 (23%)
Stable disease	18 (46%)
Progressive disease	13 (31%)

**Table 3:** Number of patients with treatment-related toxicity in the safety population (*n* = 40).

Toxicity (NCI-CTCAE), no. (%)	Grade 1	Grade 2	Grade 3	Grade 4	Any
Neutropenia	5 (12.5%)	10 (25%)	15 (37.5%)	1 (2.5%)	31 (77.5%)
Thrombocytopenia	4 (10%)	15 (37.5%)	6 (15%)	1 (2.5%)	26 (65%)
Anemia	13 (32.5%)	15 (37.5%)	10 (25%)	0	38 (95%)
Alopecia	6 (15%)	1 (2.5%)	0	0	7 (17.5%)
Diarrhea	10 (25%)	10 (25%)	0	0	20 (50%)
Nausea/vomiting	15 (37.5%)	5 (12.5%)	1 (2.5%)	0	21 (52.5%)
Neurotoxicity	14 (35%)	2 (5%)	8 (20%)	0	24 (60%)
Nephrotoxicity	10 (25%)	4 (10%)	6 (15%)	0	20 (50%)
Hepatotoxicity	10 (25%)	5 (12.5%)	6 (15%)	0	21 (52.5%)

NCI-CTCAE indicates National Cancer Institute Common Terminology Criteria for Adverse Events.
